# Supplementary material for: Tumor cell-directed STING agonist antibody-drug conjugates induce type III interferons and anti-tumor innate immune responses
Source: Nat Commun. 2024 Jul 11;15:5842. doi: 10.1038/s41467-024-49932-4 (PMC11239908; doi:10.1038/s41467-024-49932-4)
Supplement: Supplementary file 1 — Supplementary Information [file 41467_2024_49932_MOESM1_ESM.pdf]

Tumor Cell-Directed STING Agonist Antibody-Drug Conjugates Induce Type III Interferons and Anti-Tumor Innate Immune Responses

Naniye Malli Cetinbas[1]*, Travis Monnell[1], Jahna Soomer-James[1], Pamela Shaw[1], Kelly Lancaster[1], Kalli C. Catcott[1], Melissa Dolan[1], Rebecca Mosher[1], Caitlin Routhier[1], Chen-Ni Chin[1], Dorin Toader[1], Jeremy Duvall[1], Raghida Bukhalid[1], Timothy B. Lowinger[1], Marc Damelin[1]*

[1]Mersana Therapeutics Inc. Cambridge MA 02139, USA

*Corresponding Authors (ncetinbas@mersana.com, mdamelin@mersana.com)

SUPPLEMENTARY INFORMATION

- **Supplementary Figures 1-22 and Supplementary Figure Legends**
- **Supplementary Table 1**
- **Supplementary References**

SUPPLEMNTARY FIGURES

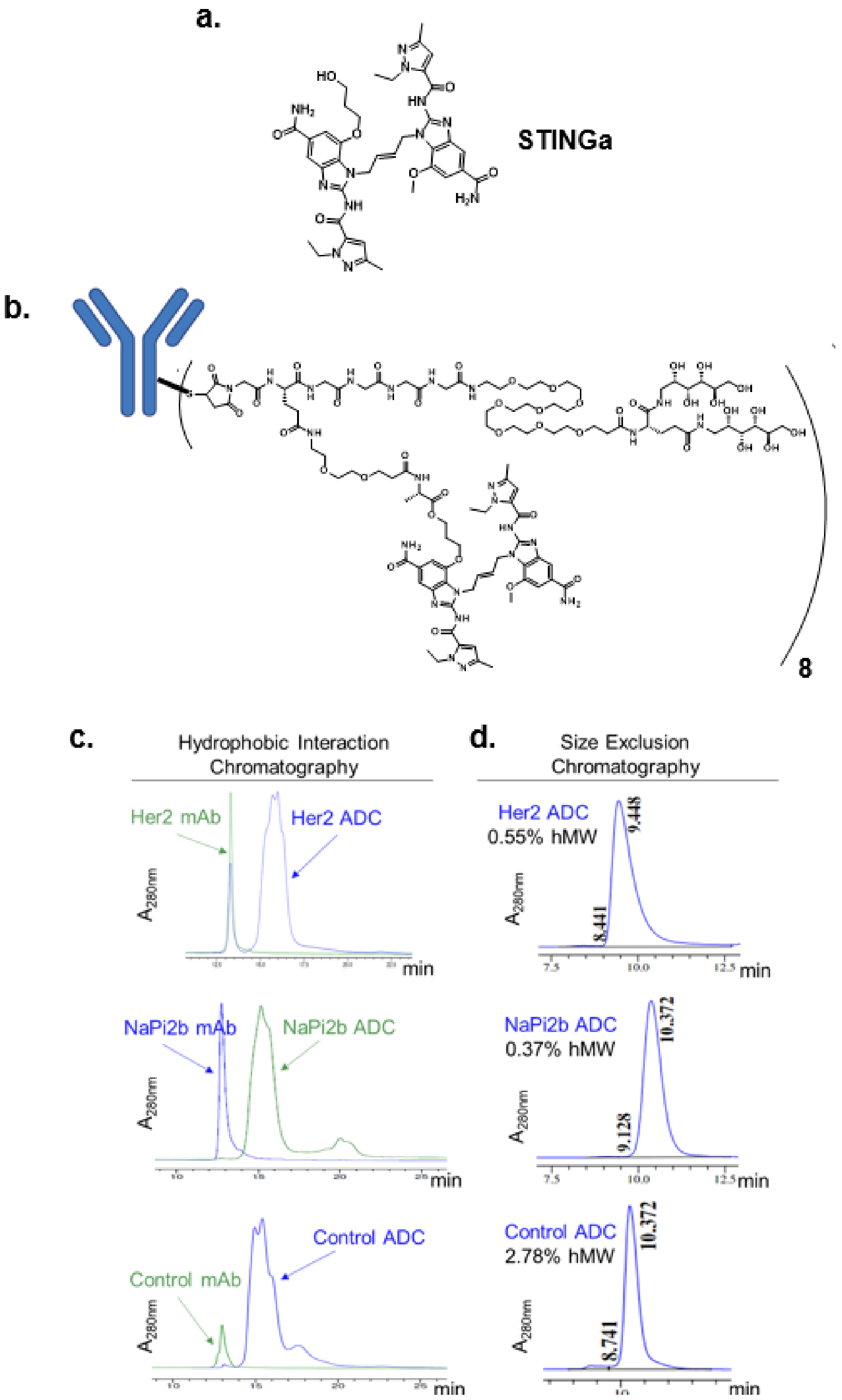

Supplementary Figure 1. Generation of STINGa ADCs. a. Structure of the payload STINGa. **b.** A representative schematic of STINGa-ADCs. STINGa-ADCs were prepared by reducing the interchain disulfide bonds of the antibody with TCEP followed by conjugation to the payload with thiol-maleimide chemistry. **c.** Hydrophobic interactions chromatography of the ADCs plotted with parental antibodies overlaid. **d.** Size exclusion chromatography of the ADCs confirming minimal aggregation.

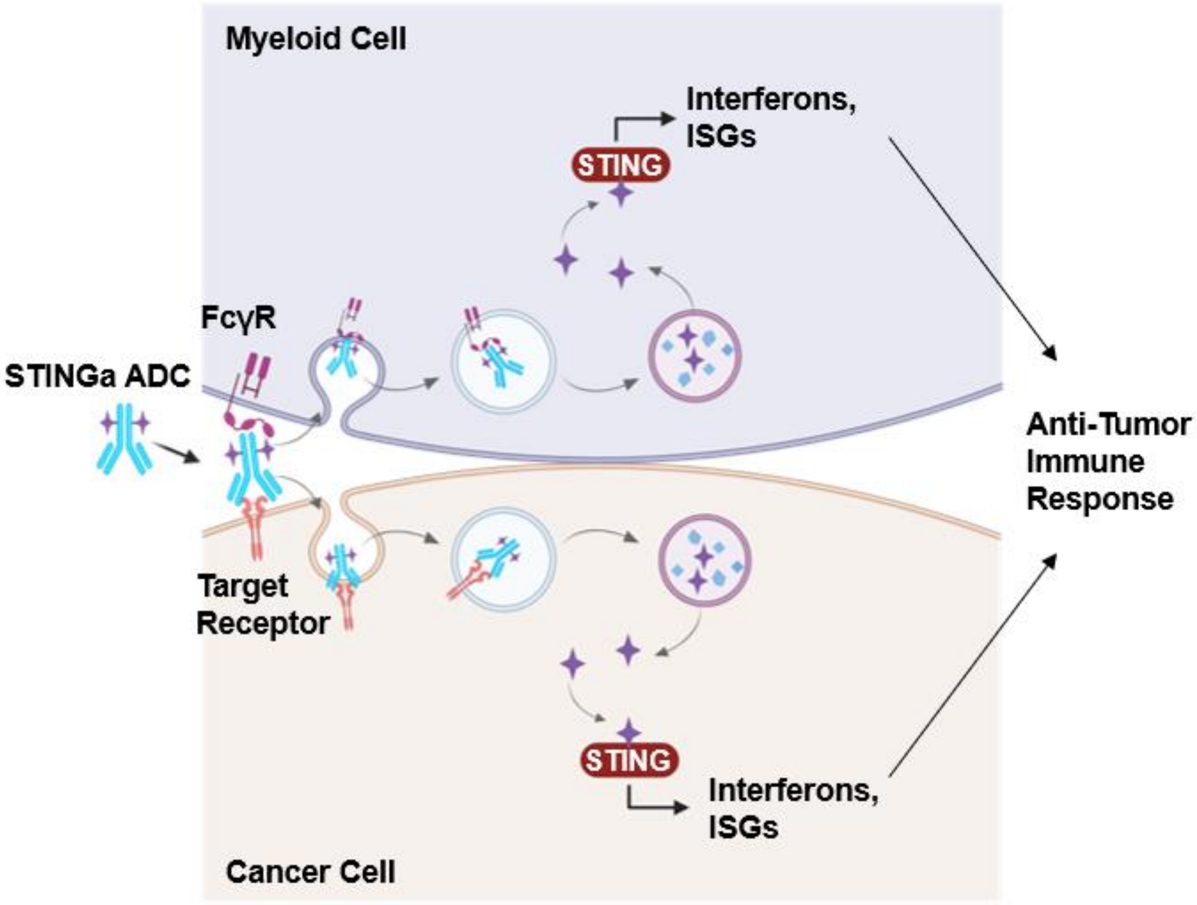

Supplementary Figure 2. Cartoon representation of a tumor cell-directed STINGa-ADC internalization and processing by cancer cells and myeloid cells leading to anti-tumor immune responses. Cartoon was generated using biorender.com.

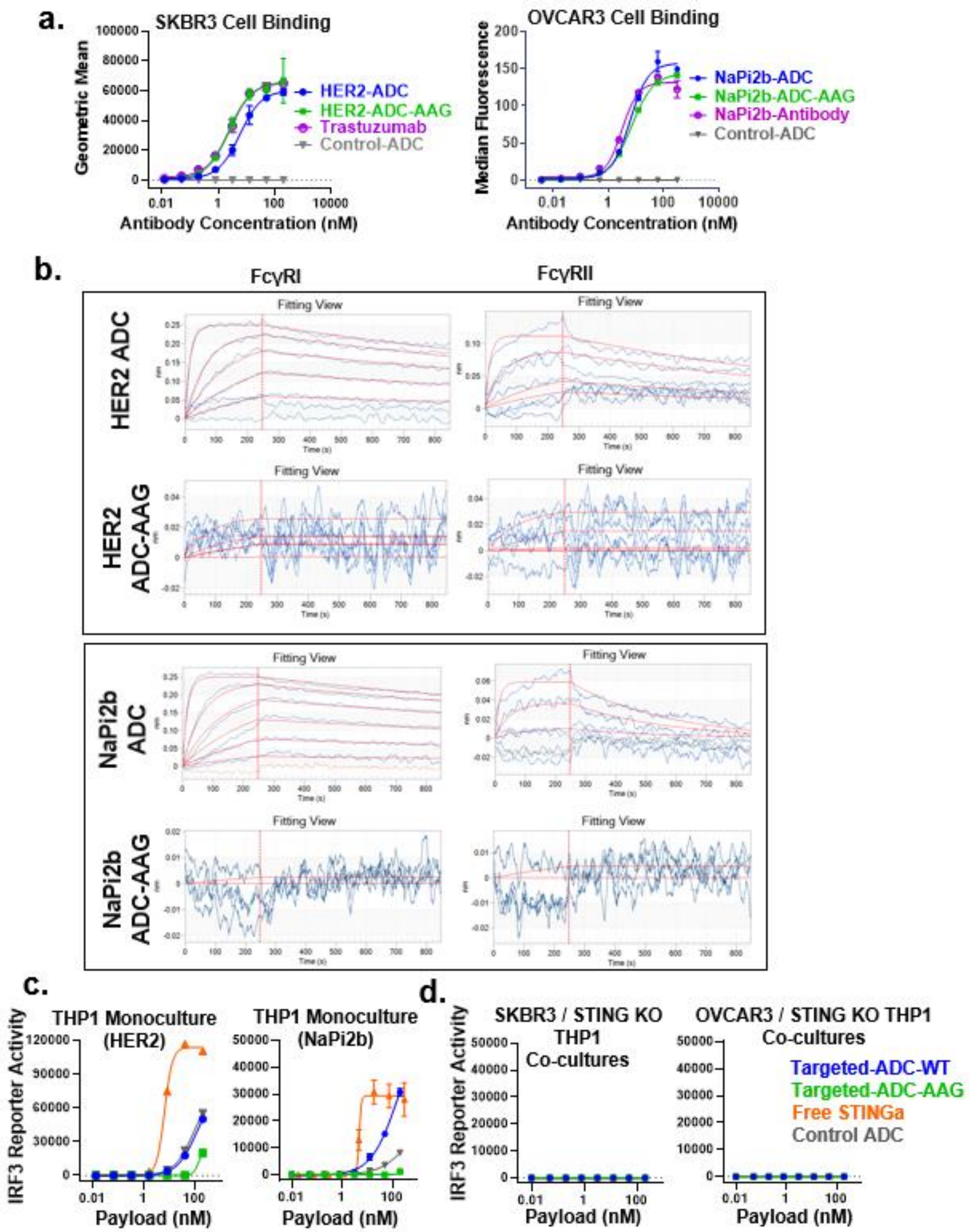

Supplementary Figure 3. a. Dose response curves for binding of the Fc-wt, Fc-mutant HER2- or NaPi2b STING-ADCs, unconjugated antibodies, and the Control-ADC to the SKBR3 (HER2) and OVCAR3 (NaPi2b) cells respectively, measured by flow cytometry. **b.** Graphical traces of binding kinetics of Fc wt and AAG mutant HER2 and NaPi2b STING ADCs to human Fcγ-RI and Fcγ-RII recombinant protein, showing association and dissociation phases of binding as measured by Bio-Layer Interferometry assay. n=4 technical replicates, data is representative of at least two independent experiments. **c.** Graphs showing IRF3 reporter activity in THP monocultures in the absence of target antigen 24 hours after indicated treatments. **d.** Graphs showing IRF3 reporter activity in STING KO THP1 reporter cells co-cultured with SKBR3 (HER2) or OVCAR3 (NaPi2b) cells 24 hours after the indicated treatments. Data shown in **a, c, d** are mean ± SD (n=2 biological replicates) and representative of two independent experiments. Source data are provided as a Source Data file.

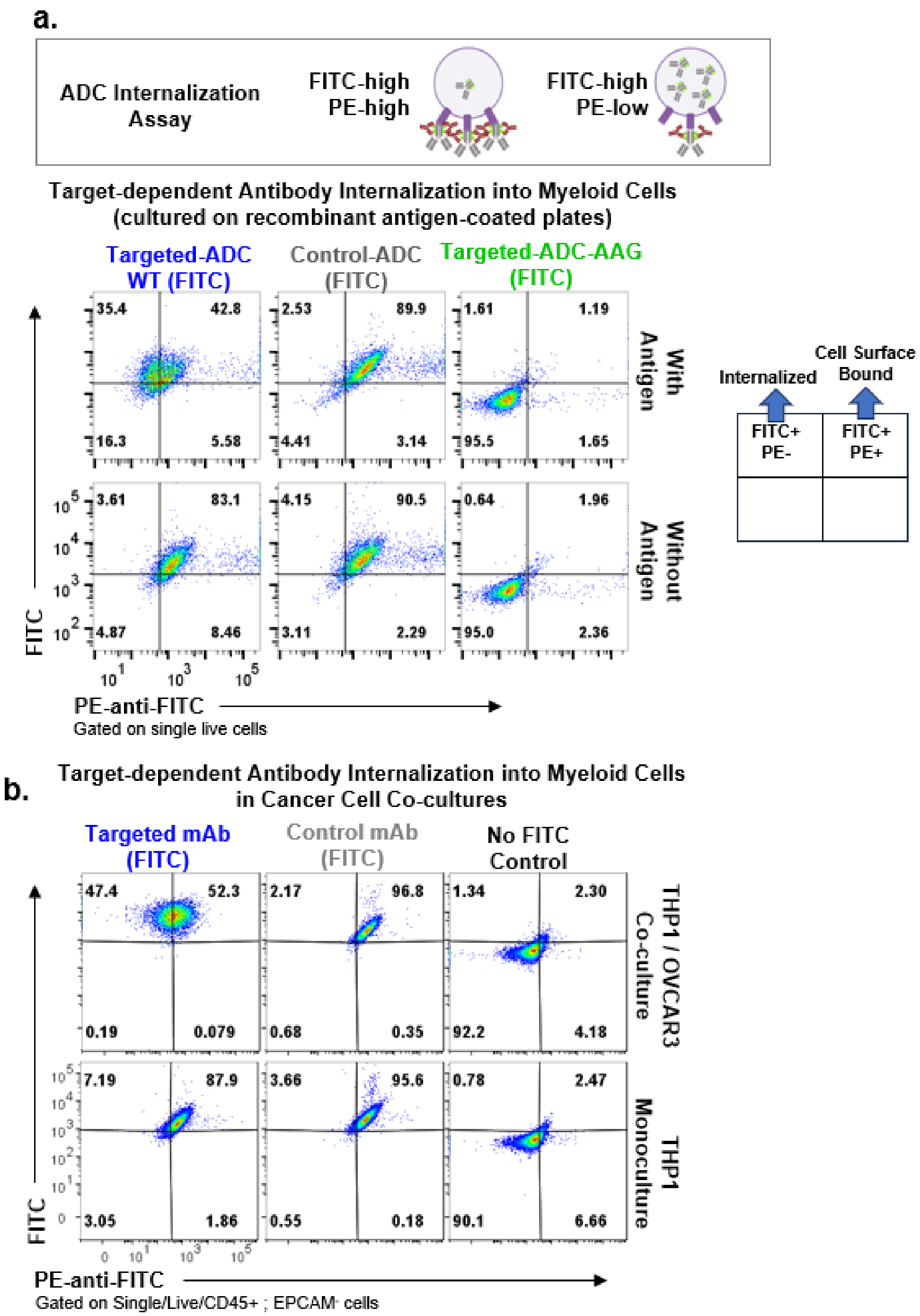

Supplementary Figure 4. a. Flow cytometry analysis of PE-anti-FITC-stained THP1 myeloid cells, which were cultured on recombinant NaPi2b-coated plates or non-coated control plates in the presence of FITC-conjugated STINGa-ADCs (20 nM based on payload) for 6 hours. **b,** Flow cytometry analysis of the cell surface bound (FITC+ PE+) vs internalized (FITC+ PE-) FITC-conjugated targeted antibody (NaPi2b) or non-binding control antibody by flow cytometry using the anti-FITC antibody (PE) in THP1 (myeloid) cell monocultures vs OVCAR3 (NaPi2b+ cancer cell) co-cultures (T=5 hours). Data demonstrates that the targeted antibody is internalized into THP1 myeloid cells in the presence of target-expressing cancer cells. Data shown in **a-b** are mean ± SD (n=3 biological replicates) and representative of two independent experiments. Source data are provided as a Source Data file.

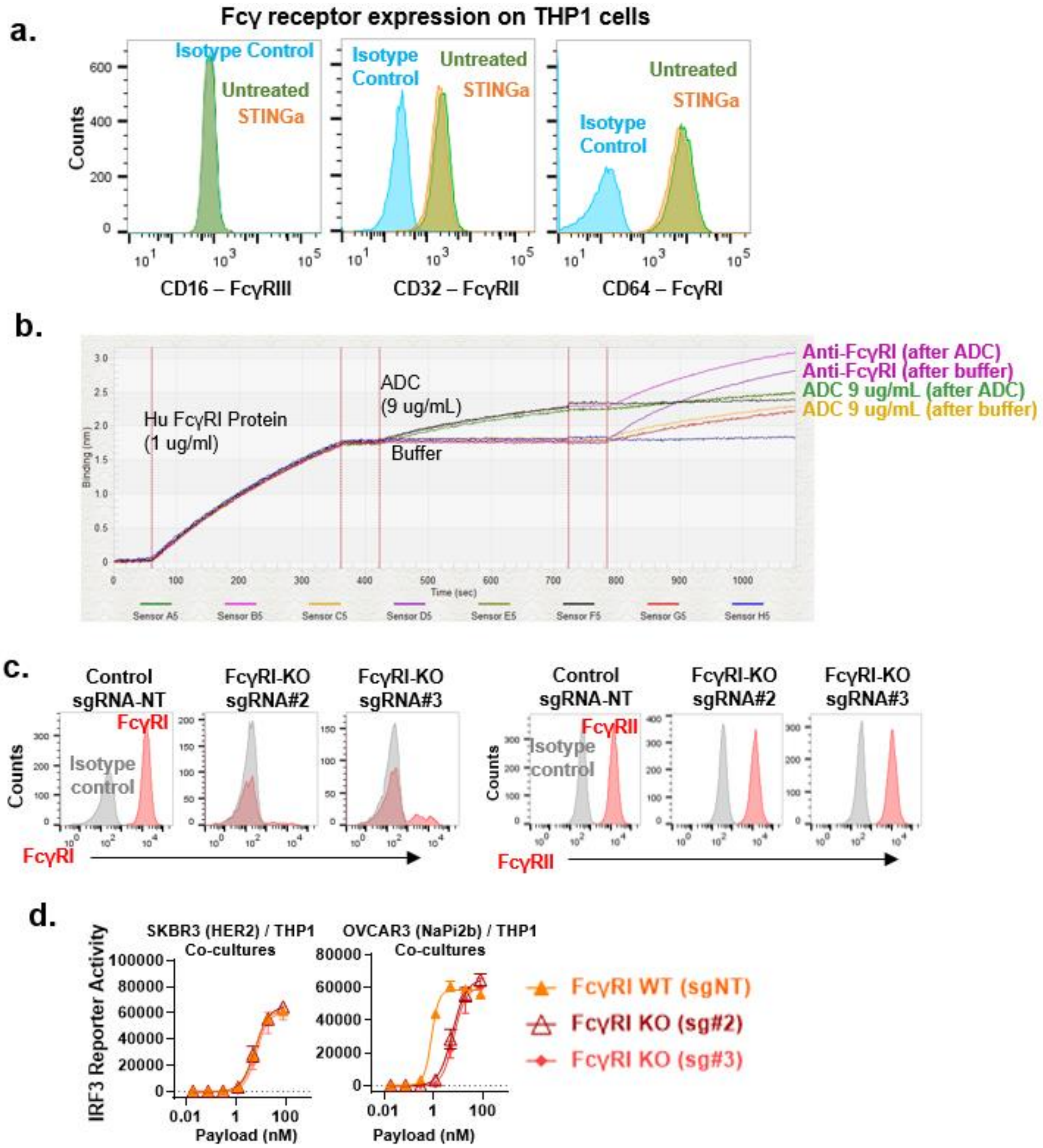

Supplementary Figure 5. a. Flow cytometry analysis of FcγRIII, FcγRII, and FcγRI expression on THP1 cells with/without STINGa treatment demonstrating that the receptor expression levels do not change following STINGa treatment. Data shown are representatives of three biological replicates. Experiment performed once. **b.** Human FcγRI-binding competition between NaPi2b-STINGa with wt Fc and anti-human FcγRI fluorescent antibody, which was used to detect cell surface expression of FcγRI on THP1 cells (Fig. 1i) was performed by Biolayer Interferometry assay. Data demonstrates that the anti-FcγRI antibody can bind to human FcγRI following ADC-Fc-binding. Traces are average of three technical replicates. Experiment performed once. **c.** Flow cytometry analysis of FcγRI and FcγRII, expression on wild type (sgRNA-NT) or FcγRI knock out (sgRNA#2 and sgRNA#3) THP1 cells. Data shown are representatives of three technical replicates. FcγRI expression analysis was repeated independently. **d.** Graphs showing IRF3 reporter activity in FcγRI wild type (WT) or knock our (KO) THP1 reporter cells co-cultured with SKBR3 (HER2) or OVCAR3 (NaPi2b) cells in the presence of increasing concentrations of free STINGa payload (T=24 hours, n=3 biological replicates). Data shown is representative of two independent experiments. Source data are provided as a Source Data file.

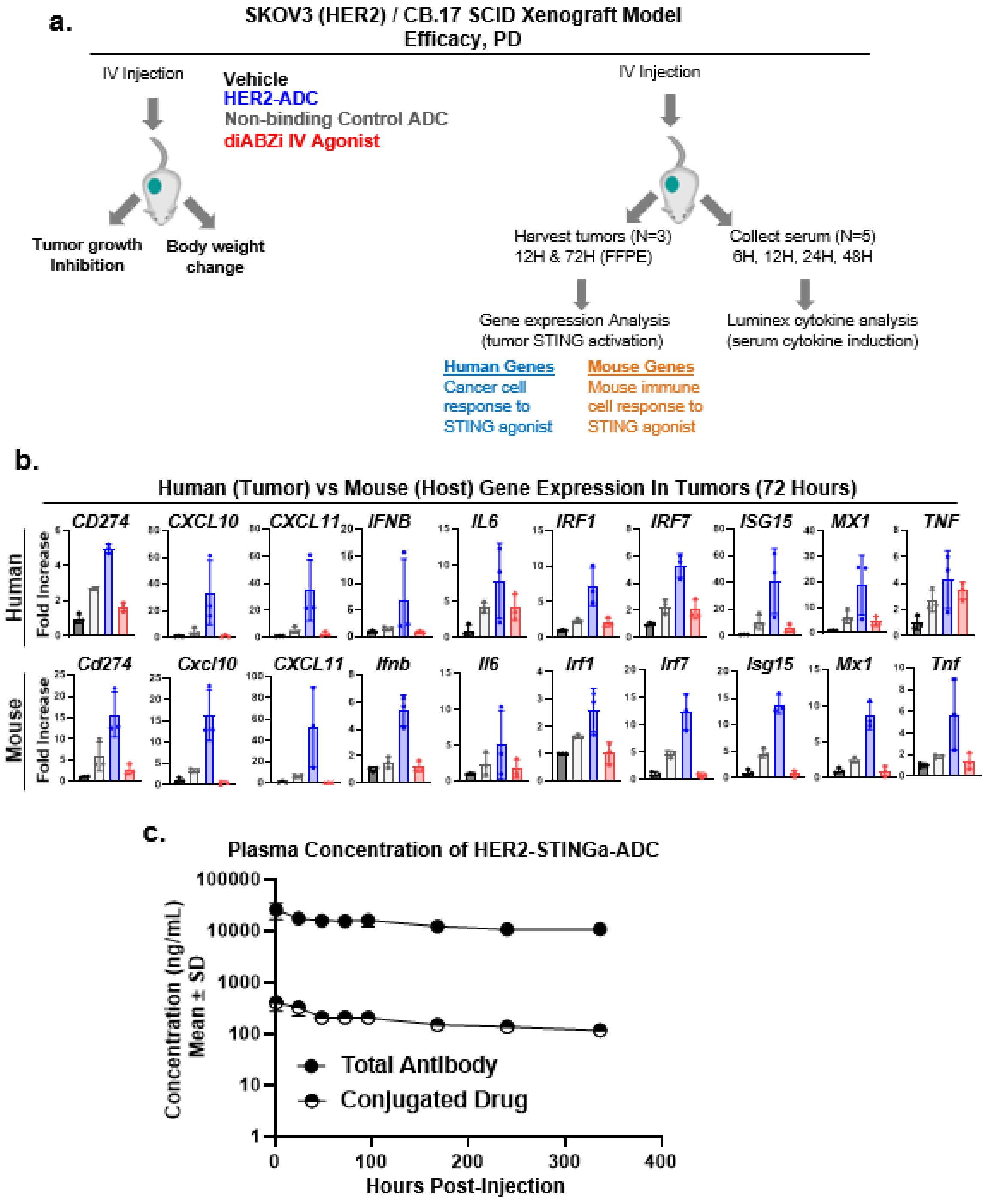

Supplementary Figure 6. a. Schematic of the SKOV3 / SCID efficacy and PD study that was performed in parallel (Fig. 2) to determine the systemic cytokine vs tumor STING pathway activation. **b.** Bar graphs show fold increases in human or mouse STING pathway genes in tumors treated with the indicated test articles (T=72 hours). Data shown are mean ± SD (n=3 mice/group). **c.** Total antibody and antibody-conjugated drug concentrations of the HER2-STINGa-ADC in the plasma samples collected from non-tumor bearing naïve SCID mice after treatment with 3/0.1 mg/kg (mAb/payload) were measured and plotted against time. Data shown are mean ± SD (n=3 mice/group). Source data are provided as a Source Data file.

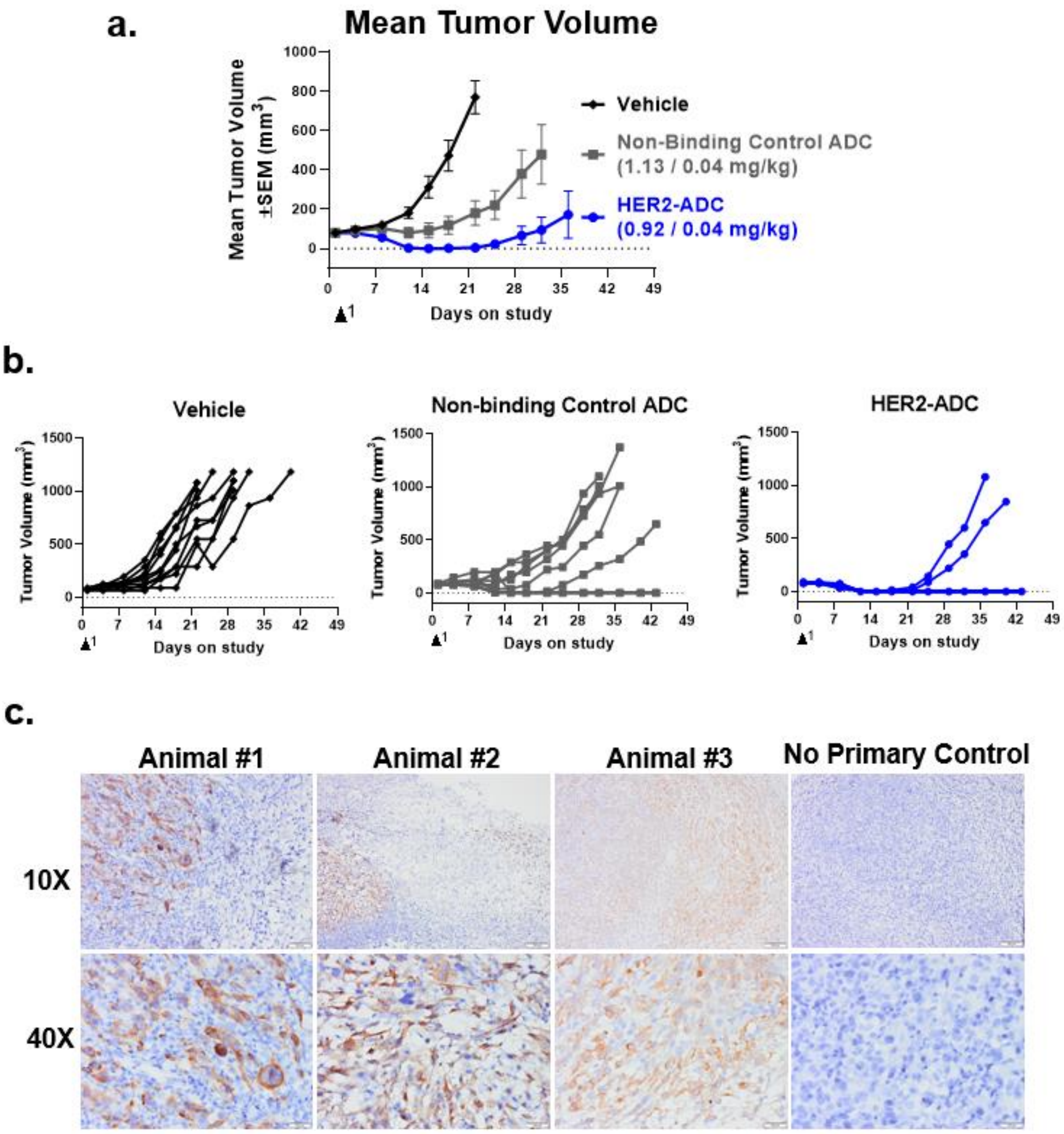

Supplementary Figure 7. a. Efficacy of the single IV dose of the HER2-ADC and the non-binding Control-ADC in the 4T1-humanHER2-engineered syngeneic tumor model in BALB/c mice. Doses shown are antibody/payload. Data shown are mean tumor volume ± SEM (N=10 mice/group; arrow shows day 1 of dosing). **b.** Tumor growth curves of the individual animals treated as in (**a**). **c.** HER2 expression in the engineered 4T1-Human HER2 tumors in Balb/C by Immunohistochemistry, showing both membranous and cytoplasmic expression pattern that is heterogenous. IHC images are representative. Scale bars: 100 µm. Source Data are provided as a Source Data file.

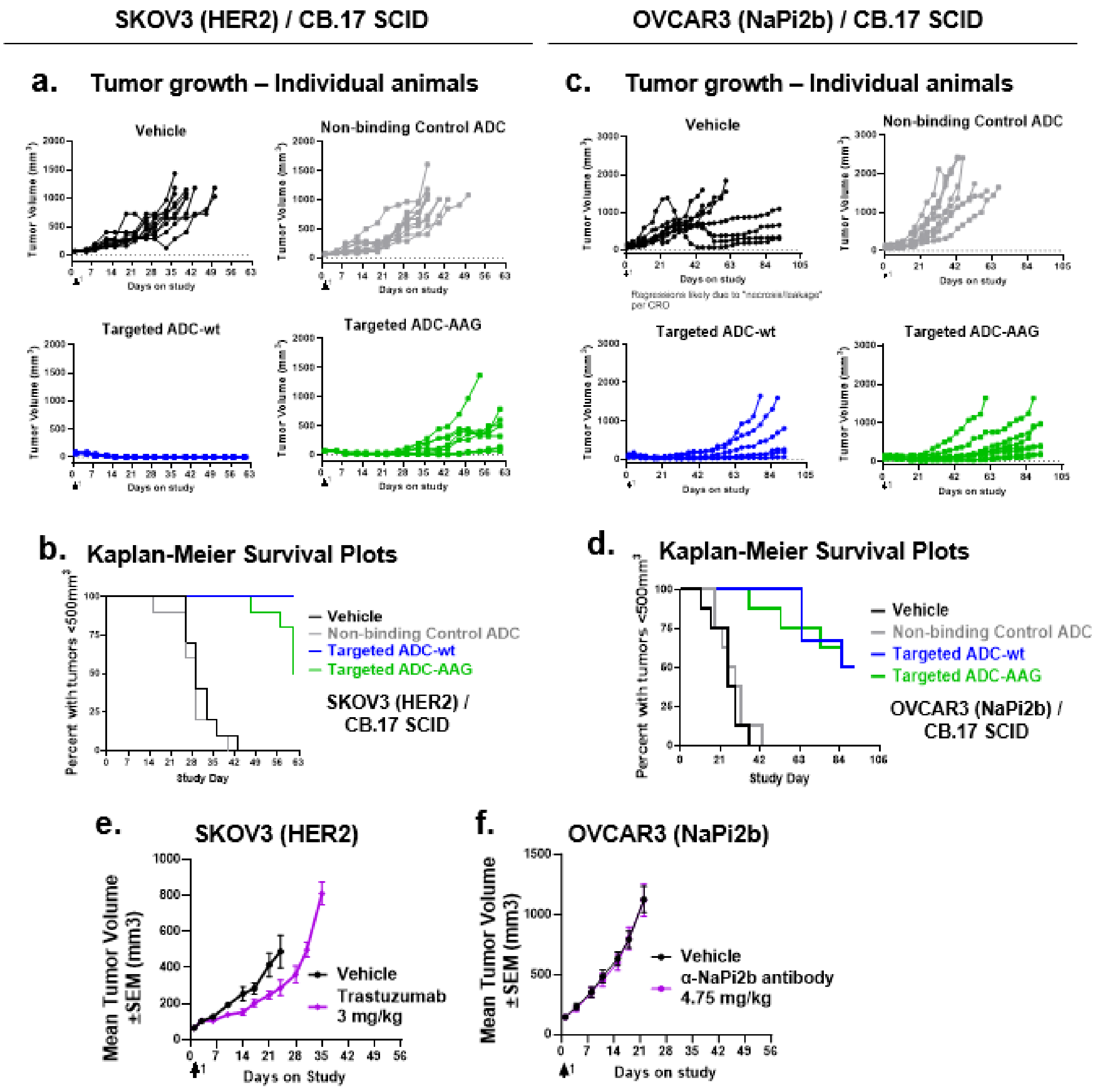

Supplementary Figure 8. Individual growth curves of **a.** SKOV3 (HER2) (n=10 mice/group) and **b.** OVCAR3 (NaPi2b) (n=8 mice/group) human tumor xenografts in CB.17 SCID mice after a single dose of vehicle, HER2 ADC-wt (3 / 0.1 mg/kg), HER2 ADC-AAG (3 / 0.1 mg/kg), and non-binding control ADC (3 / 0.1 mg/kg). ADC doses are by antibody / payload (STINGa). Kaplan-Myer survival plots for the **c.** SKOV3 and **d.** OVCAR3 tumor-bearing mice treated as indicated. Growth of **e.** SKOV3 (HER2) and **f.** OVCAR3 (NaPi2b) human tumor xenografts in SCID mice after a single IV dose of unconjugated anti-HER2 antibody (Trastuzumab) or anti-NaPi2b antibodies respectively at the indicated doses. Data points (**e, f**) show mean tumor volumes ± SEM (n=10 mice/group; arrow shows day of dosing). Source Data are provided as a Source Data file.

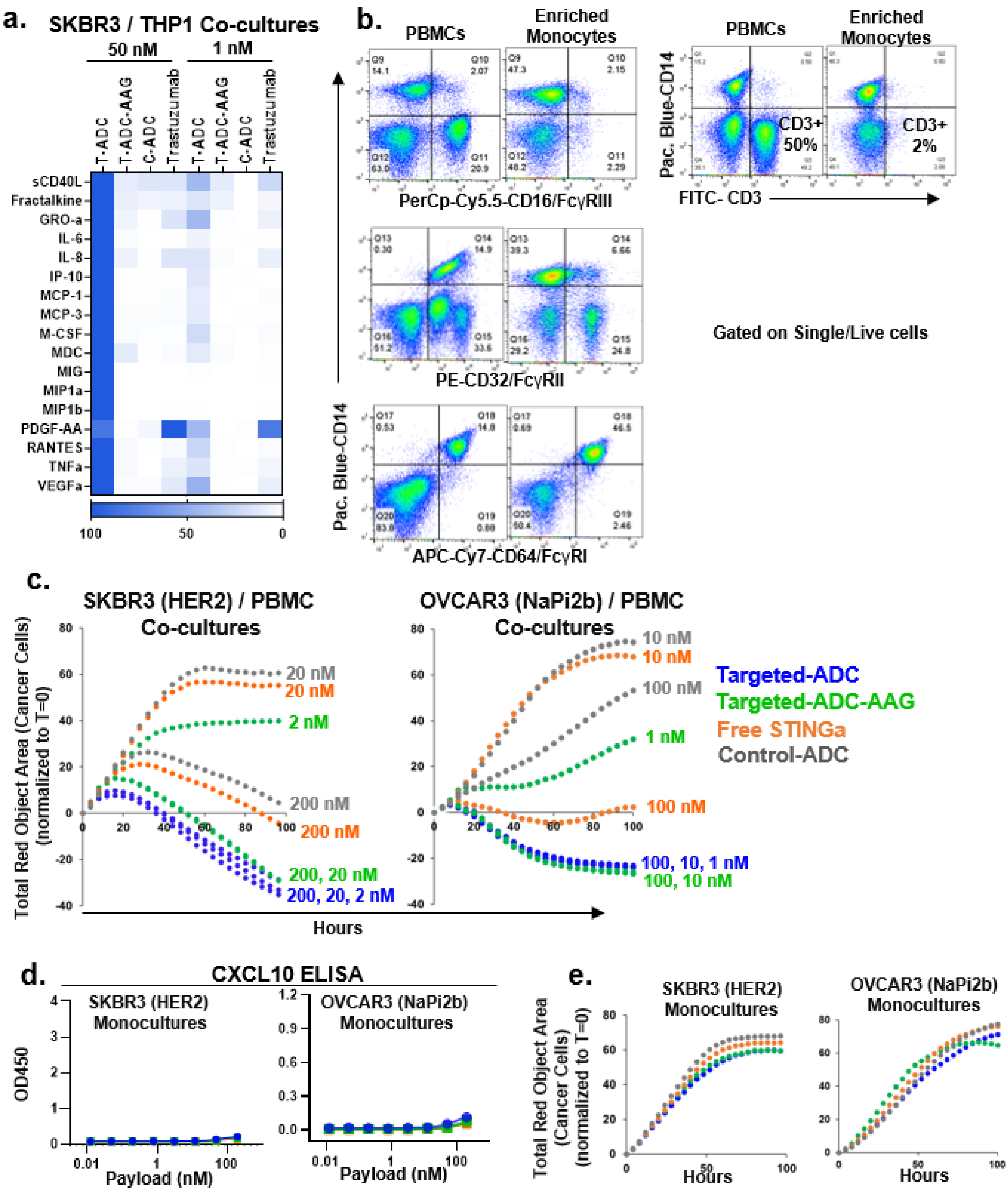

Supplementary Figure 9. Tumor cell-specific delivery of the STING agonist leads to anti-tumor activity in cancer cell and PBMC co-cultures. a. Heat map of cytokines induced in SKBR3 (HER2) cancer cell and THP1 co-culture supernatants 24 hours after treatment with 50 nM or 1 nM (based on payload) HER2 ADC-wt, HER2 ADC-AAG, non-binding control-ADC, and unconjugated anti-HER2 antibody (trastuzumab) (antibody dose equivalent of HER2-ADC-wt) measured by a multiplex cytokine assay. The scale bar shows the normalized intensity generated by GraphPad Prism software. Normalization was done for each cytokine (average of three biological replicates) (highest and lowest values were considered 100 and 0 respectively). Analysis was performed once. **b.** Flow cytometry analysis of CD14 vs CD3, FcγRI,

FcγRII, or FcγRIII expression on PBMCs and enriched monocytes. Data shown are representatives of three independent (technical) replicates. **c.** Growth curves of SKBR3 (HER2)-NR or OVCAR3 (NaPi2b)-NR cells in PBMC co-cultures treated with the indicated test articles. Percent red object area (cancer cells) was obtained from IncuCyte analysis and plotted against time. **d.** Dose response curves for CXCL10 cytokine production in SKBR3 (HER2)-NR or OVCAR3 (NaPi2b)-NR cells in monocultures 24 hours post-treatment with the indicated test articles. **e.** Graphs showing percent red object area as a measure of growth of SKBR3 (HER2) or OVCAR3 (NaPi2b) cells over time in the presence of indicated treatments (200 nM based on payload) in monocultures. Red object area in each well was normalized to T=0 values at each time point. Data points shown in the graphs in **(c - e)** are mean ± SD of three biological replicates. Source Data are provided as a Source Data file. Results shown in **(b - e)** are representatives of two independent experiments. Source data are provided as a Source Data File.

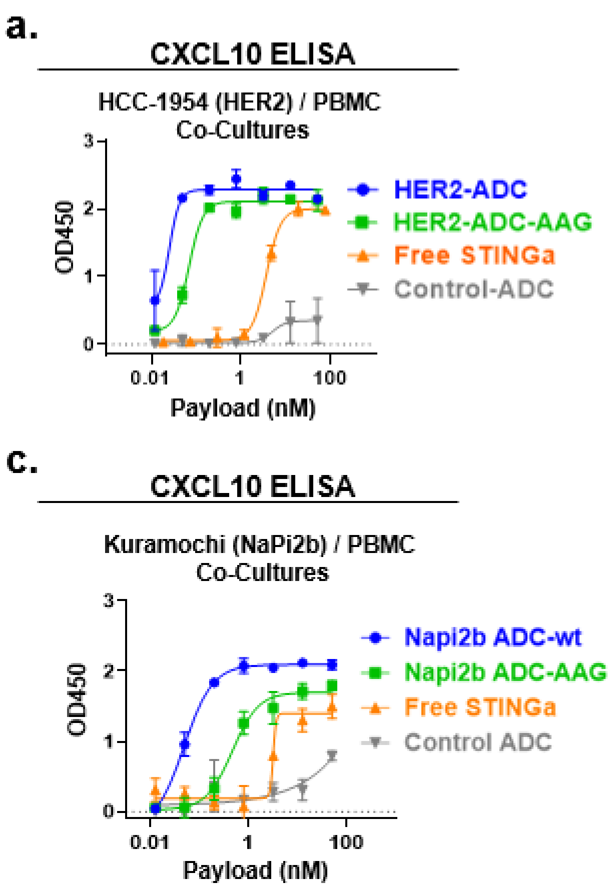

Supplementary Figure 10. a. Dose response curves for CXCL10 cytokine production in HCC-1954 (HER2) cells in co-cultures with PBMCs 24 hours post-treatment with the indicated test articles. **b.** Flow cytometry analysis of HER2 expression on HCC-1954 cells. **c.** Dose response curves for CXCL10 cytokine production in Kuramochi (NaPi2b) cells in co-cultures with PBMCs 24 hours post-treatment with the indicated test articles. **d.** Flow cytometry analysis of NaPi2b expression on Kuramochi cells. Target expression levels were determined as described in the Cell-Binding Assay under the Methods section. Data shown in **(a, c)** are mean ± SD of three biological replicates and representative of two independent experiments. Data shown in **(b, d)** are mean ± SD of three independent technical replicates and representatives of two independent experiments. Source data are provided as a Source Data file.

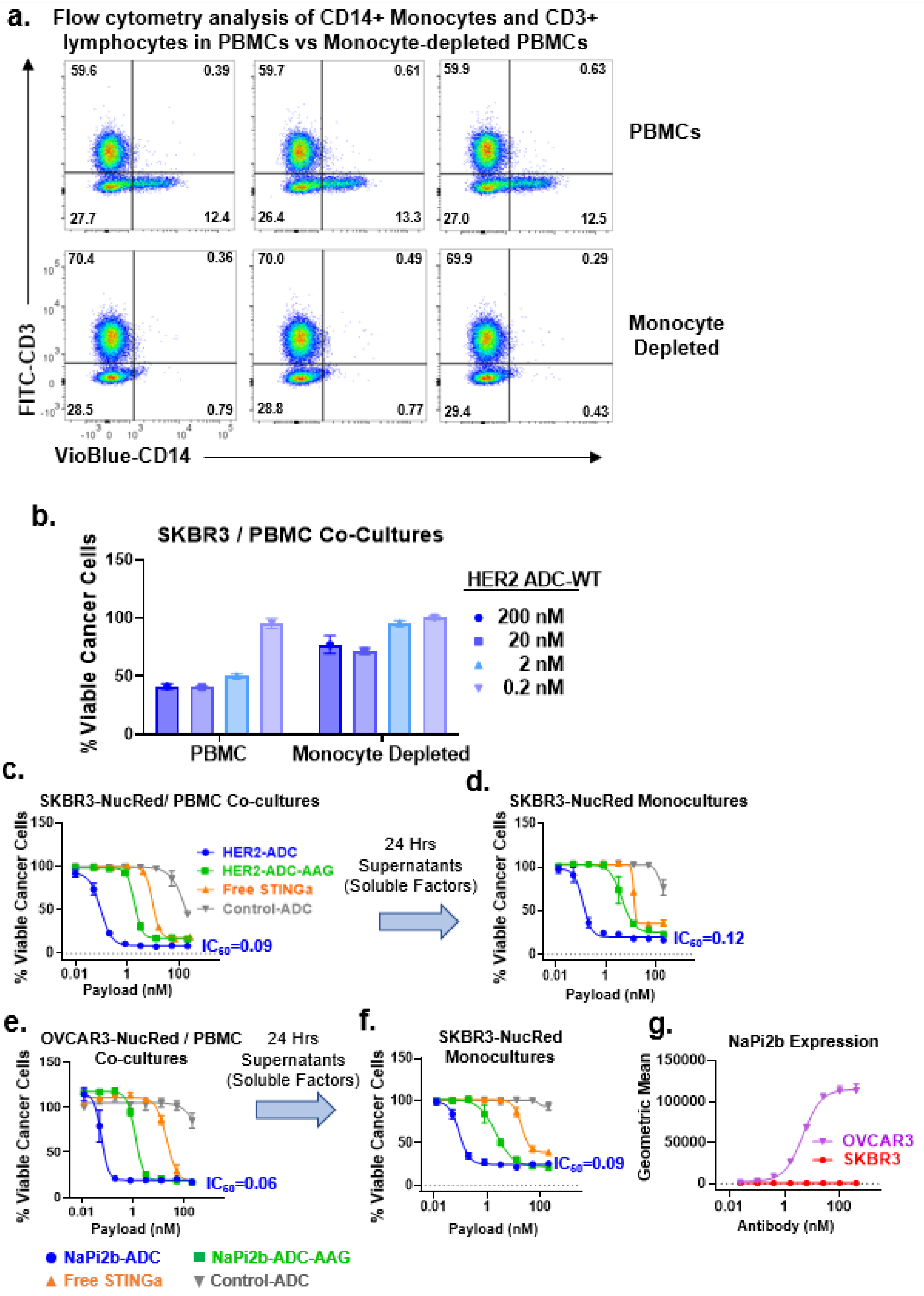
a. Flow cytometry analysis of CD14+ Monocytes and CD3+ lymphocytes in PBMCs vs Monocyte-depleted PBMCs
59.6
0.39
27.7
12.4
59.7
0.61
26.4
13.3
59.9
0.63
27.0
12.5
PBMCs
70.4
0.36
28.5
0.79
70.0
0.49
28.8
0.77
69.9
0.29
29.4
0.43
Monocyte Depleted
FITC-CD3
VioBlue-CD14
b. SKBR3 / PBMC Co-Cultures
% Viable Cancer Cells
HER2 ADC-WT
200 nM
20 nM
2 nM
0.2 nM
PBMC
Monocyte Depleted
c. SKBR3-NucRed/ PBMC Co-cultures
HER2-ADC
HER2-ADC-AAG
Free STINGa
Control-ADC
IC50=0.09
Payload (nM)
24 Hrs Supernatants (Soluble Factors)
d. SKBR3-NucRed Monocultures
IC50=0.12
e. OVCAR3-NucRed / PBMC Co-cultures
IC50=0.06
24 Hrs Supernatants (Soluble Factors)
f. SKBR3-NucRed Monocultures
IC50=0.09
g. NaPi2b Expression
Geometric Mean
OVCAR3
SKBR3
Antibody (nM)
NaPi2b-ADC
NaPi2b-ADC-AAG
Free STINGa
Control-ADC

Supplementary Figure 11. a. Flow cytometry analysis of CD3 vs CD14 expression on PBMCs or monocyte-depleted PBMCs (three independent samples, gated on single/live cells). Data is representative of three independent technical replicates. **b.** Bar graphs showing the viability of SKBR3-NR cells co-cultured with either PBMCs or monocyte-depleted PBMCs treated with the HER2-ADC at the indicated doses for 84 hours. Percent viability was calculated based on the viability of the vehicle-treated condition. **c.** Dose response curves for the viability of the SKBR3-NR cells in PBMC co-cultures treated with the indicated test articles (T=84 hours). **d.** Dose response curves for the viability of the SKBR3-NR cells in mono-cultures treated with the supernatants collected from the sister plates treated for 24 hours as in (**c**). **e.** Dose response curves for the viability of the OVCAR3-NR cells in PBMC co-cultures treated with the indicated test articles (T=84 hours). **f.** Dose response curves for the viability of the SKBR3-NR cells in mono-cultures treated with the supernatants collected from the sister plates treated for 24 hours as in (**e**). Percent viability was calculated based on the viability of the untreated condition. Data shown in **(b - f)** are mean ± SD (n= 3 biological replicates). **g.** Dose response curves for the binding of the anti-Napi2b antibody to OVCAR3 and SKBR3 as determined by flow cytometry showing lack of NaPi2b expression on SKBR3 cells. Data points are mean ± SD (n= 3 technical replicates). Data shown in **(a-g)** are representatives of two independent experiments. Source data are provided as a Source Data file.

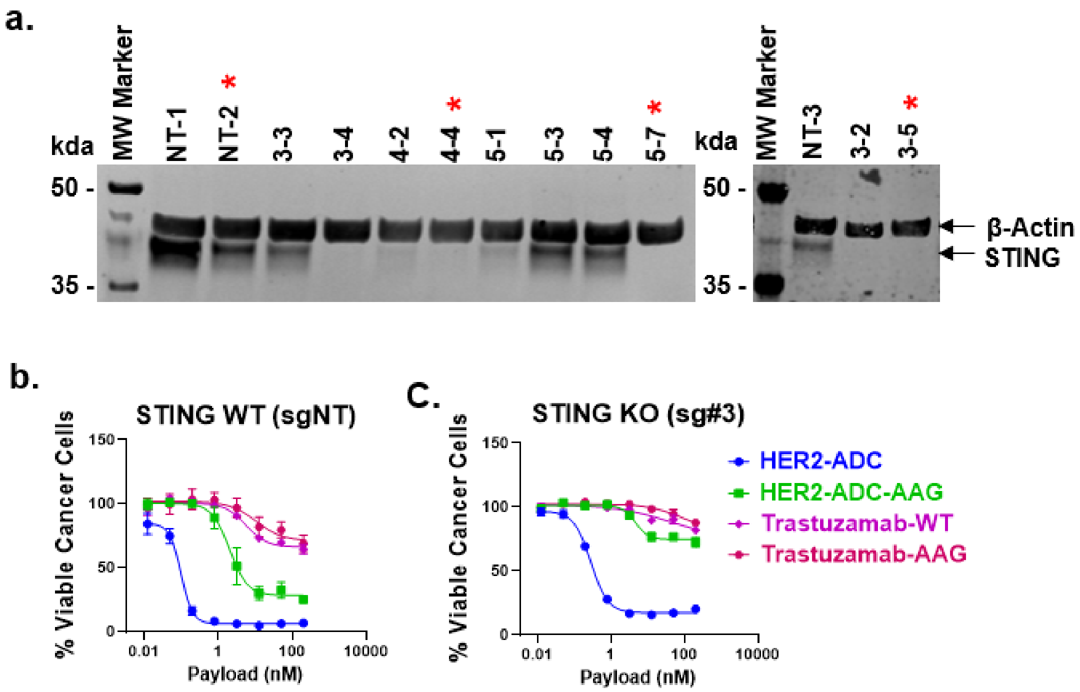

Supplementary Figure 12. a. Western blot analysis of STING protein expression in SKBR3 single cell clones generated using a non-targeting control sgRNA, and three unique sgRNAs against *STING* gene. β-Actin was used as a loading control. Clones that were selected for functional assays are denoted by a star in red font. MW Marker: molecular weight marker. Images are from n=1 sample for each cell line and representative of two independent analyses. Dose response curves for the viability of the SKBR3 NucRed **b.** STING WT and **c.** STING KO single cells clones 84 hours after treatment with Fc-wt HER2-ADC, Fc-mutant HER2-ADC, and the corresponding unconjugated parental antibodies. Red object confluency in each well at 84-hour time point was normalized to T=0 values. Percent viability was calculated based on the viability of the untreated condition. Data shown in **(b, c)** are mean ± SD of n= 3 biological replicates, representatives of two independent experiments. Source data are provided as a Source Data file.

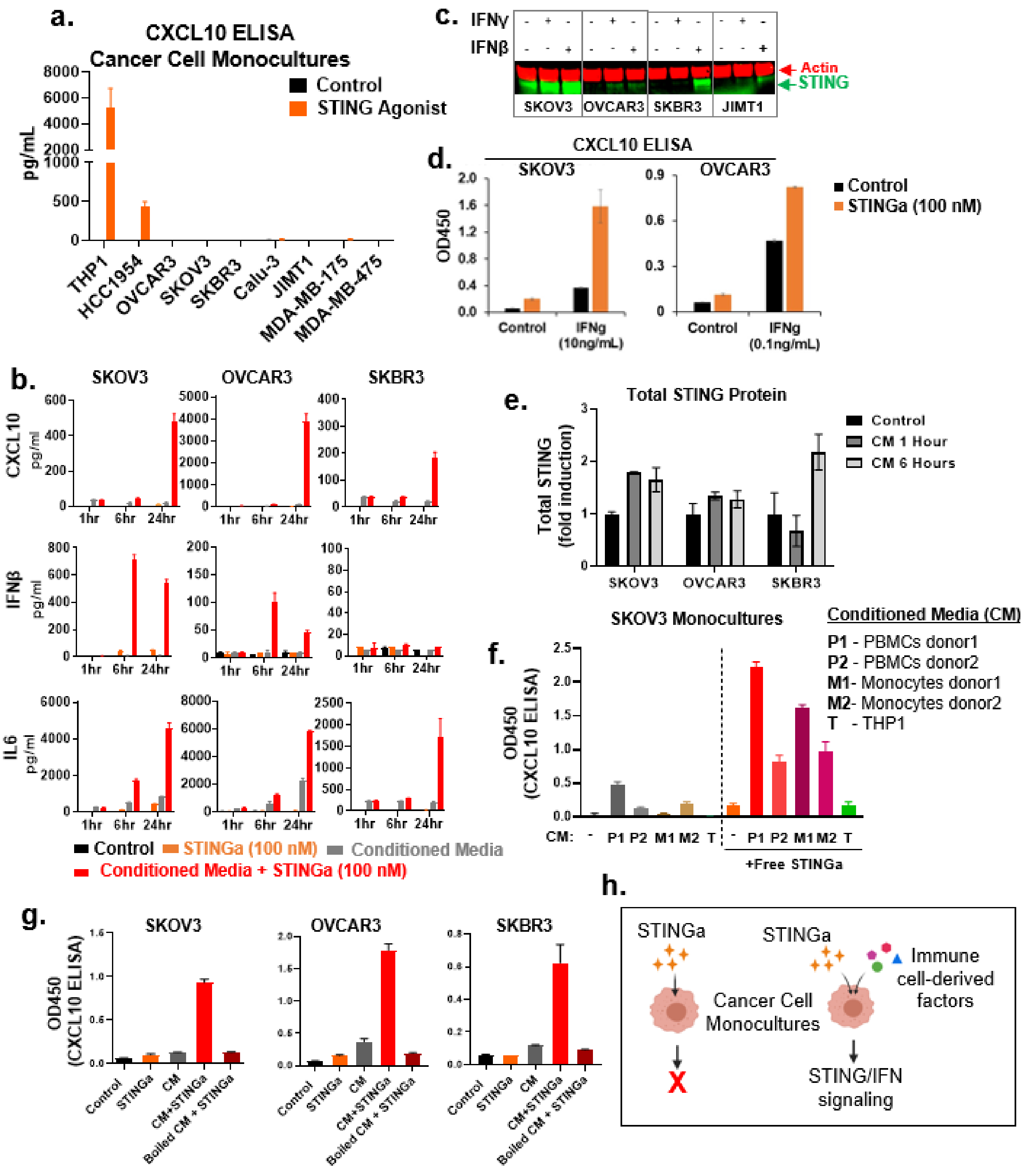

Supplementary Figure 13. a. Most cancer cell lines fail to induce CXCL10 (proxy for STING activation) in response to STINGa treatment (100 nM) in monocultures (T=24 hours). **b.** Cytokine induction in cancer cell monocultures (1, 6, 24-hour time points) treated with 100 nM STINGa alone or in the presence of conditioned media (CM) harvested from untreated human PBMC cultures (24-36 hours) measured by a multiplex Luminex assay. **c.** Western blot analysis of cancer cell monoculture lysates showing an increase in STING protein levels after treatment with recombinant IFNγ (10 or 100 ng/mL) or IFNβ (10 ng/mL) for 24 hours. Images are representative of two independent analyses (n=1 sample per condition). **d.** CXCL10 induction in the supernatants of SKOV3 and OVCAR3 monocultures by STINGa treatment (100 nM, 24 hours) measured by a human CXCL10 ELISA. **e.** Total STING protein levels (normalized to α-Tubulin) 1 hour and 6 hours after treatment with CM were determined using an HTRF-based Cisbio assay. **f.** CXCL10

induction (proxy for STING activation) in SKOV3 cells with or without STINGa treatment in the presence of CM harvested from the untreated cultures of PBMCs, isolated monocytes, or THP1 monocyte cells. **g.** CXCL10 induction is abrogated in cancer cell monocultures by STINGa treatment in the presence of boiled CM. **h.** Schematic depicting tumor cell-intrinsic STING activation in the presence of immune cell-derived factors. Data shown in **(a, b, d, e, f, g)** are mean ± SD of three biological replicates, and representatives of two independent experiments. Source data are provided as a Source Data file.

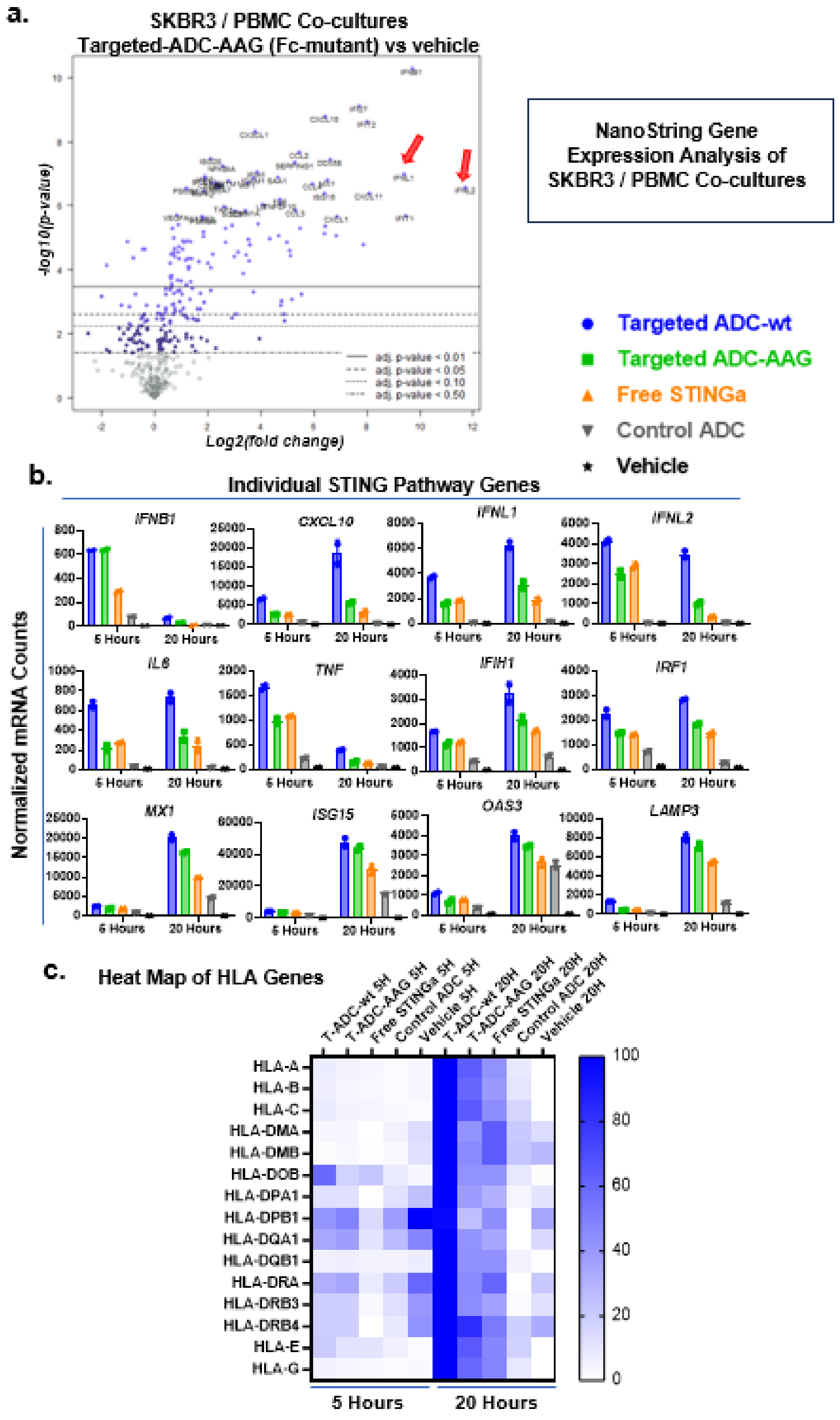

Supplementary Figure 14. a. Volcano plots showing differentially expressed genes in SKBR3 and human PBMC co-cultures treated with 50 nM (based on payload) HER2 ADC-AAG (Fc-mutant) vs vehicle for 5

hours. Arrows point to *IFNL1* and *IFNL2*. Data from n=2 biological replicates. **b.** Bar graphs showing the normalized mRNA expression of STING pathway genes in SKBR3 and human PBMC co-cultures treated with 50 nM (based on payload) of the indicated test articles for 5 hours or 20 hours. Data points shown are mean ± SD of two biological replicates. **c.** Heat map of HLA genes in the same co-cultures as in (**a** and **b**). The scale bar shows the normalized intensity generated by GraphPad Prism software. Normalization was done for mean value of each gene (n=2 biological replicates, highest and lowest normalized mRNA values were considered 100 and 0 respectively). Gene expression analysis was performed once and nSolver Advanced Analysis software was used for data analysis. Source data are provided as a Source Data file.

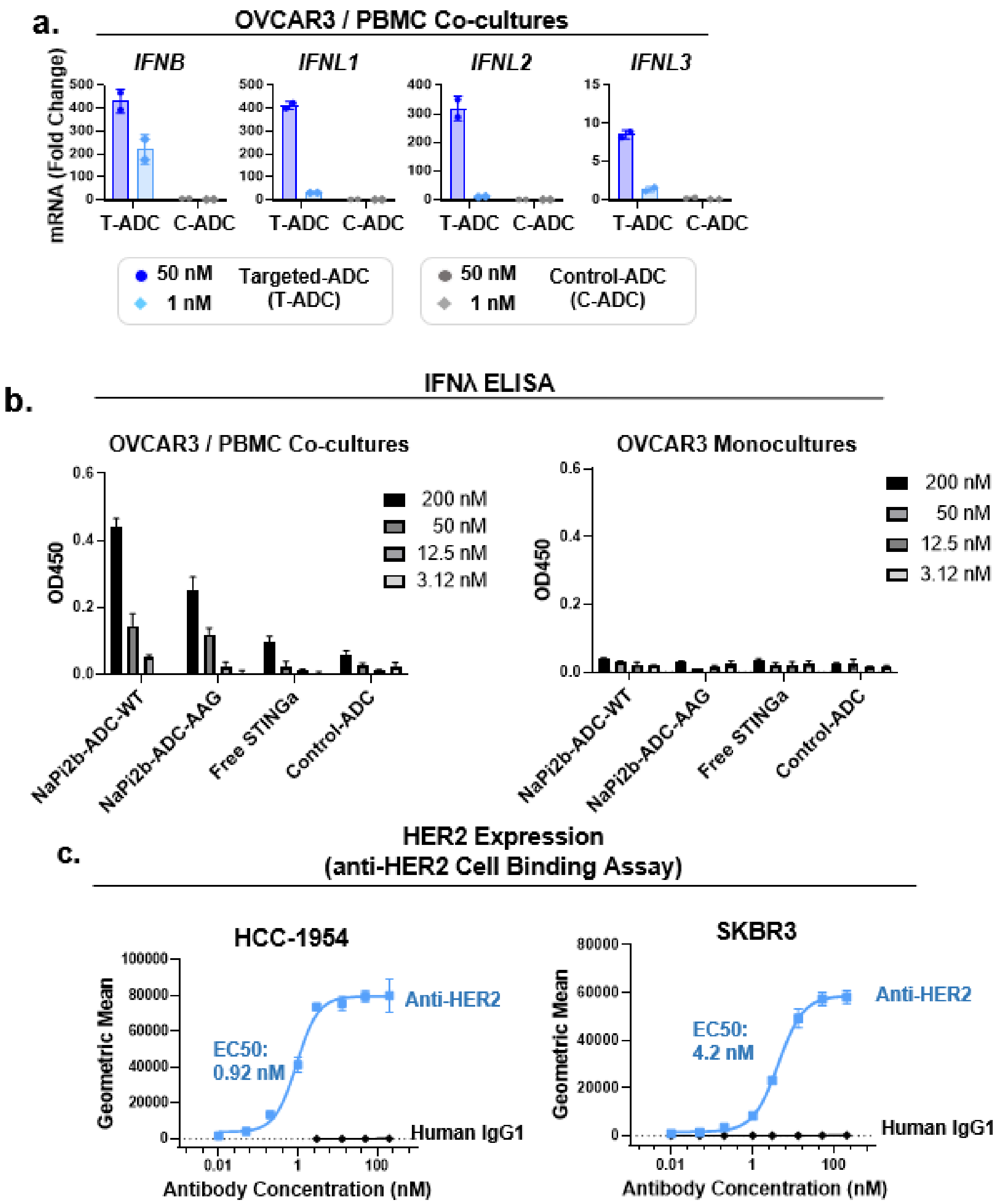

Supplementary Figure 15. a. qPCR analysis of *IFNB*, *IFNL1*, *IFNL2*, *IFNL3* mRNA expression in OVCAR3 and PBMC co-cultures treated with 50 nM or 1 nM (based on payload) NaPi2b-ADC or Control-ADC for 5 hours. mRNA was normalized to *GAPDH*. Fold changes based on the universal RNA was calculated by the ΔΔCT method). Data shown are mean ± SD of two biological replicates. Experiment performed once. **b.** IFNλ cytokine induction in OVCAR3 and PBMC co-cultures or OVCAR3 monocultures following 24 hours of treatments with Fc-wt NaPi2b-ADC, Fc-mutant (AAG) NaPi2b-ADC, free STINGa payload, and the control-ADC at the indicated doses (based on payload). Data shown are mean ± SD of three biological replicates. **c.** Dose response curves for binding of the anti-HER2 antibody (or human IgG1 control antibody) to the HCC1954 and SKBR3 cells demonstrating high HER2 expression in both cell lines. Data shown are mean ± SD of three technical replicates. Data shown in **(b, c)** are representatives of two independent experiments. Source data are provided as a Source Data file.

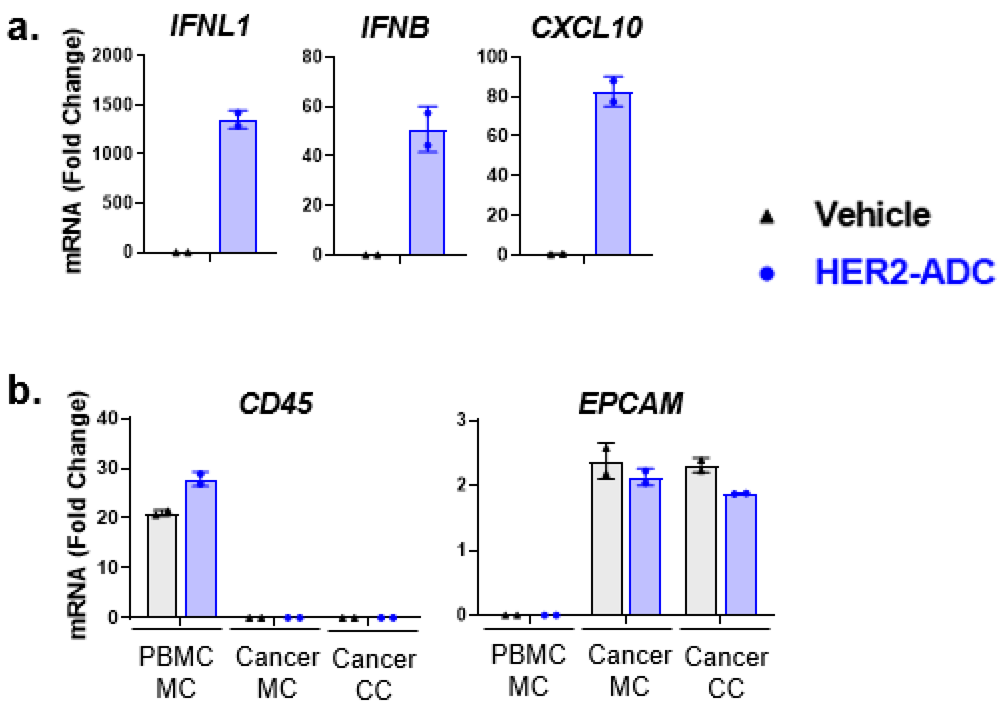

Supplementary Figure 16. qPCR analysis of **a.** *IFNB*, *IFNL1*, and *CXCL10*, **b.** *CD45* and *EPCAM* in cancer cell fractions isolated from co-cultures following 5 hours treatment with vehicle or HER2-ADC. In **b,** qPCR analysis of CD45 and EPCAM was performed on the PBMC and cancer cell monocultures to be used as controls for relative expression levels. mRNA was normalized to *GAPDH*. Fold changes based on the universal RNA was calculated by the ΔΔCT method. Data points shown in **(a, b)** are mean ± SD (n=2 biological replicates) and representatives of two independent experiments. Source data are provided as a Source Data file.

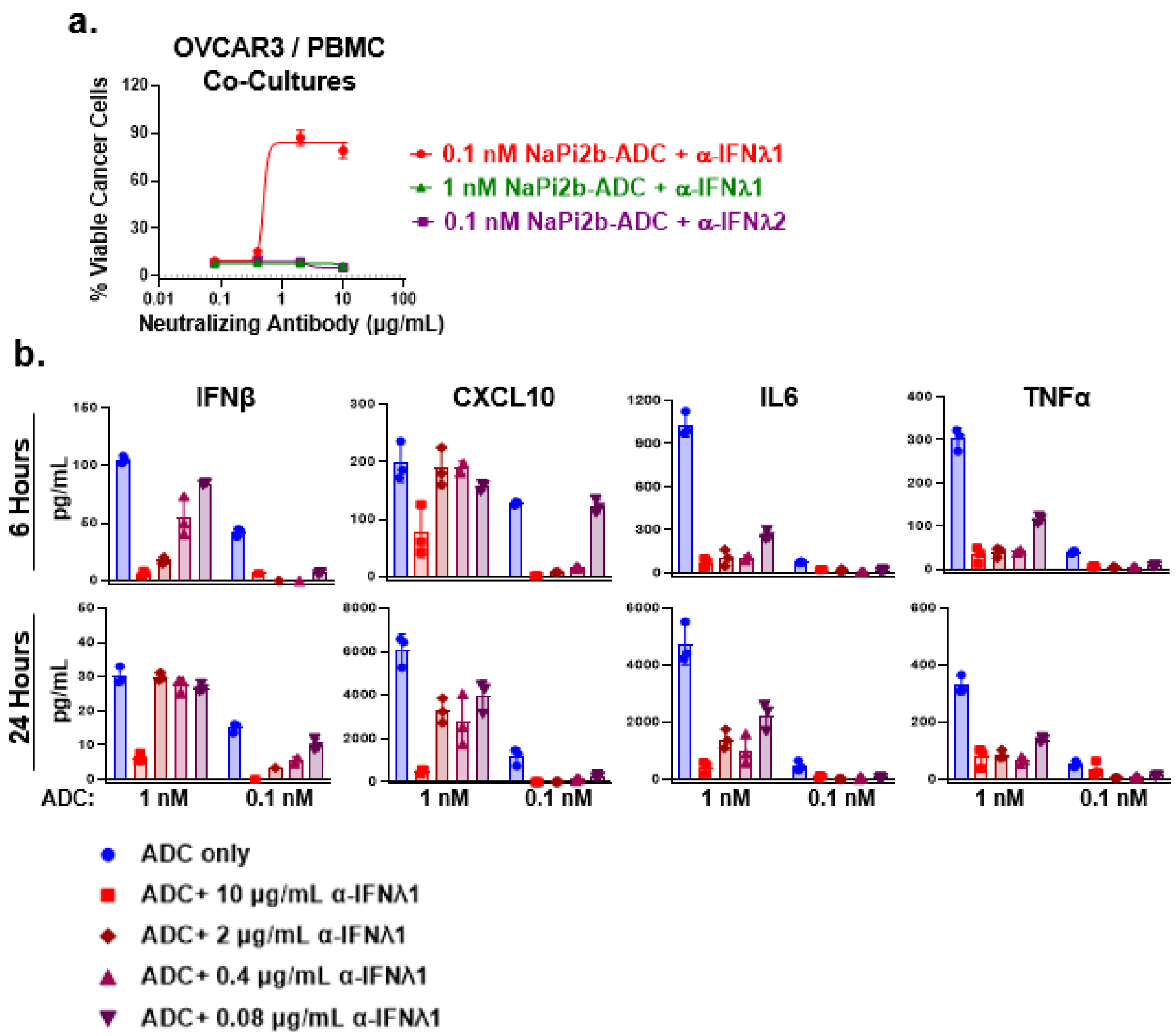

Supplementary Figure 17. a. Viability of OVCAR3 NucRed cells and PBMC co-cultures after 84 hours of treatment with 1 nM or 0.1 nM Napi2b-ADC with or without 10, 2, 0.4, 0.08 µg/mL IFNλ1 –neutralizing antibodies. A set of wells were treated with IFNλ2 –neutralizing antibodies as control. **b.** IFNβ, CXCL10, IL6, and TNFα cytokines were measured in the sister plates prepared as in **(a)** using a multiplex cytokine assay. Data shown in **(a, b)** are mean ± SD of three biological replicates and representatives of two independent experiments. Source data are provided as a Source Data file.

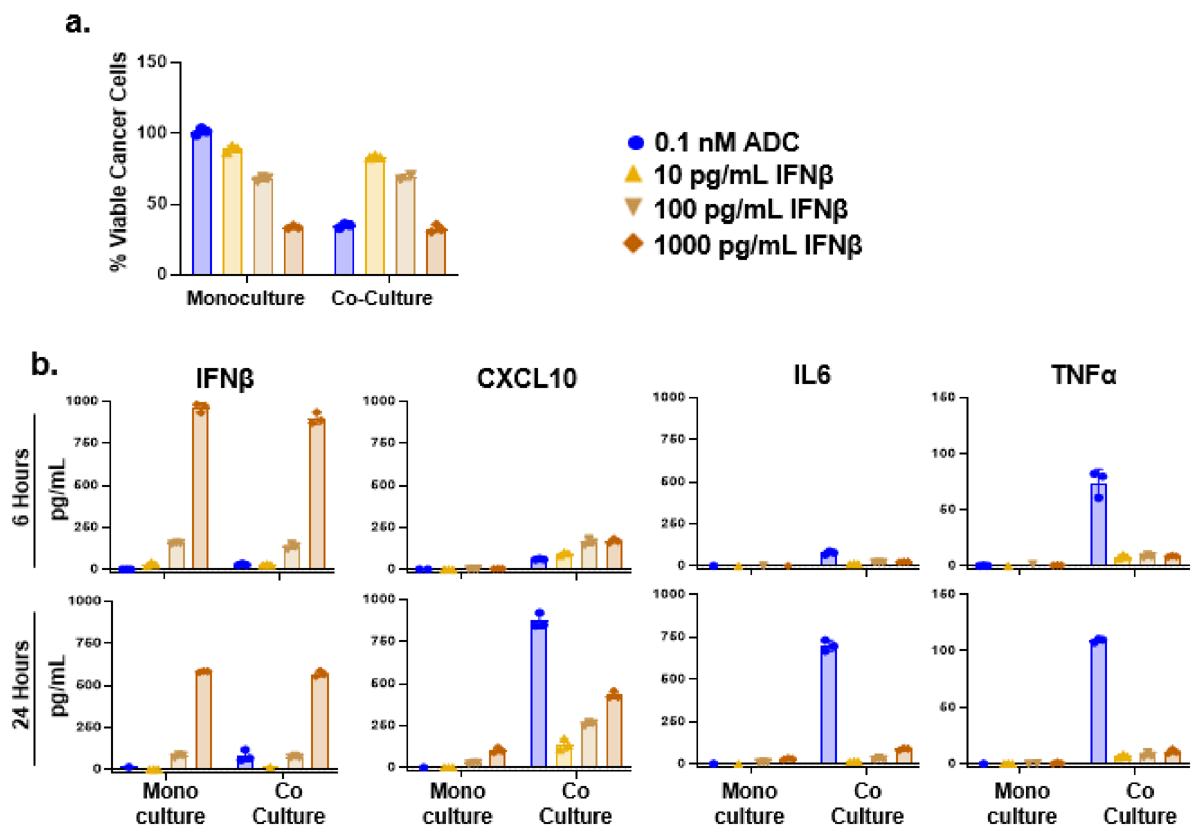

Supplementary Figure 18. a. SKBR3 NucRed cell viability in monocultures or PBMC co-cultures treated with 0.1 nM (based on payload) HER2-ADC or 1000, 100, 10 pg/mL, recombinant human IFNβ (T=84 hours). **b.** IFNβ, CXCL10, IL6, and TNFα induction in the supernatants of the sister plates as described in **(a)** after 6 hours and 24 hours of treatments using a multiplexed cytokine assay. Data shown in **(a, b)** are mean ± SD (n=3 biological replicates) and representatives of two independent experiments. Source data are provided as a Source Data file.

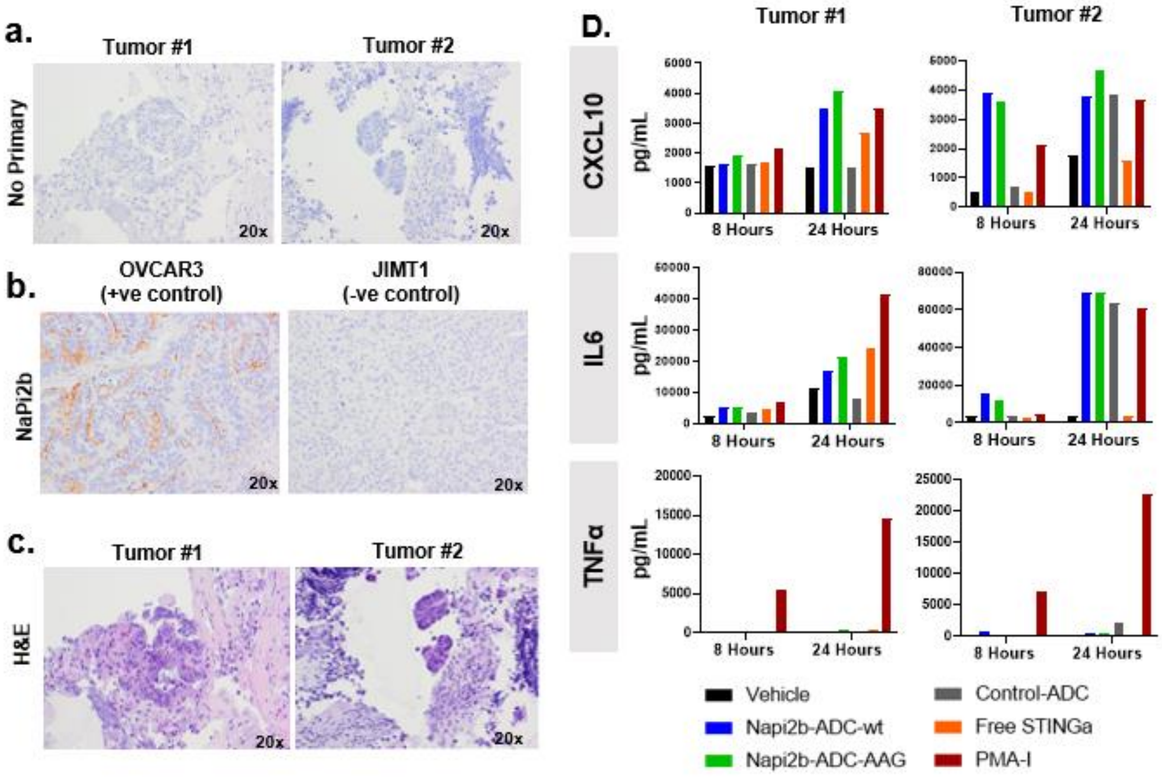

Supplementary Figure 19. a. Control IHC images of Tumor#1 and Tumor#2 stained without the primary antibody. **b.** Representative IHC images of the OVCAR3 (positive control) and JIMT1 (negative control) tumors stained with anti-NaPi2b antibody. **c.** Representative H&E images of the Tumor#1 and Tumor#2 enrolled in the ex-vivo assays. **d.** CXCL10, IL6, and TNFα cytokines were measured in the supernatants of the PDTF cultures (same samples as in Fig. 7F and Fig. 7G) after 8 hours and 24 hours of indicated treatments (20 nM based on payload) using a multiplex cytokine assay.

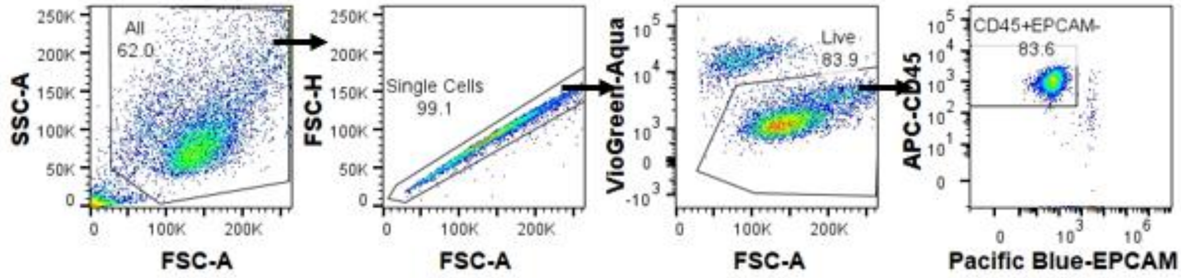

Supplementary Figure 20. Example gating strategy. From the experiment shown in Supplementary Figure 4b.

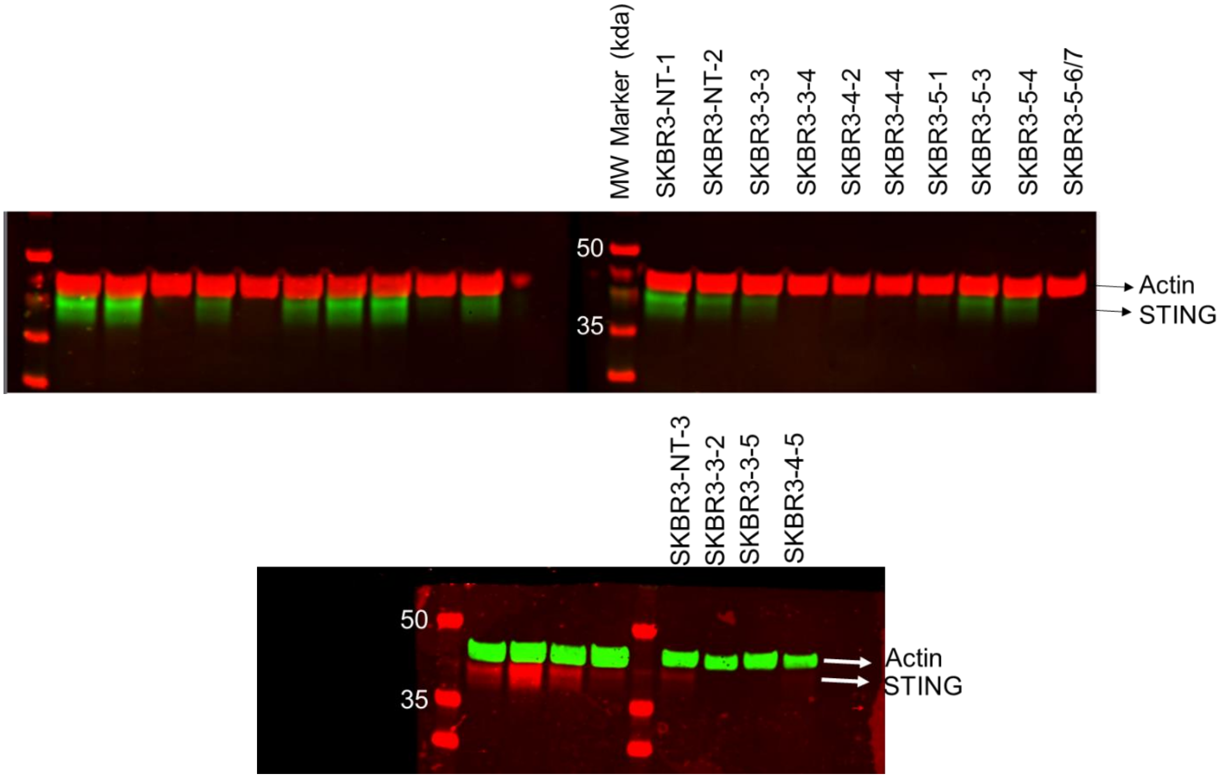

Supplementary Figure 21. Uncropped original scans of the Western blot images in Supplementary Figure 12a.

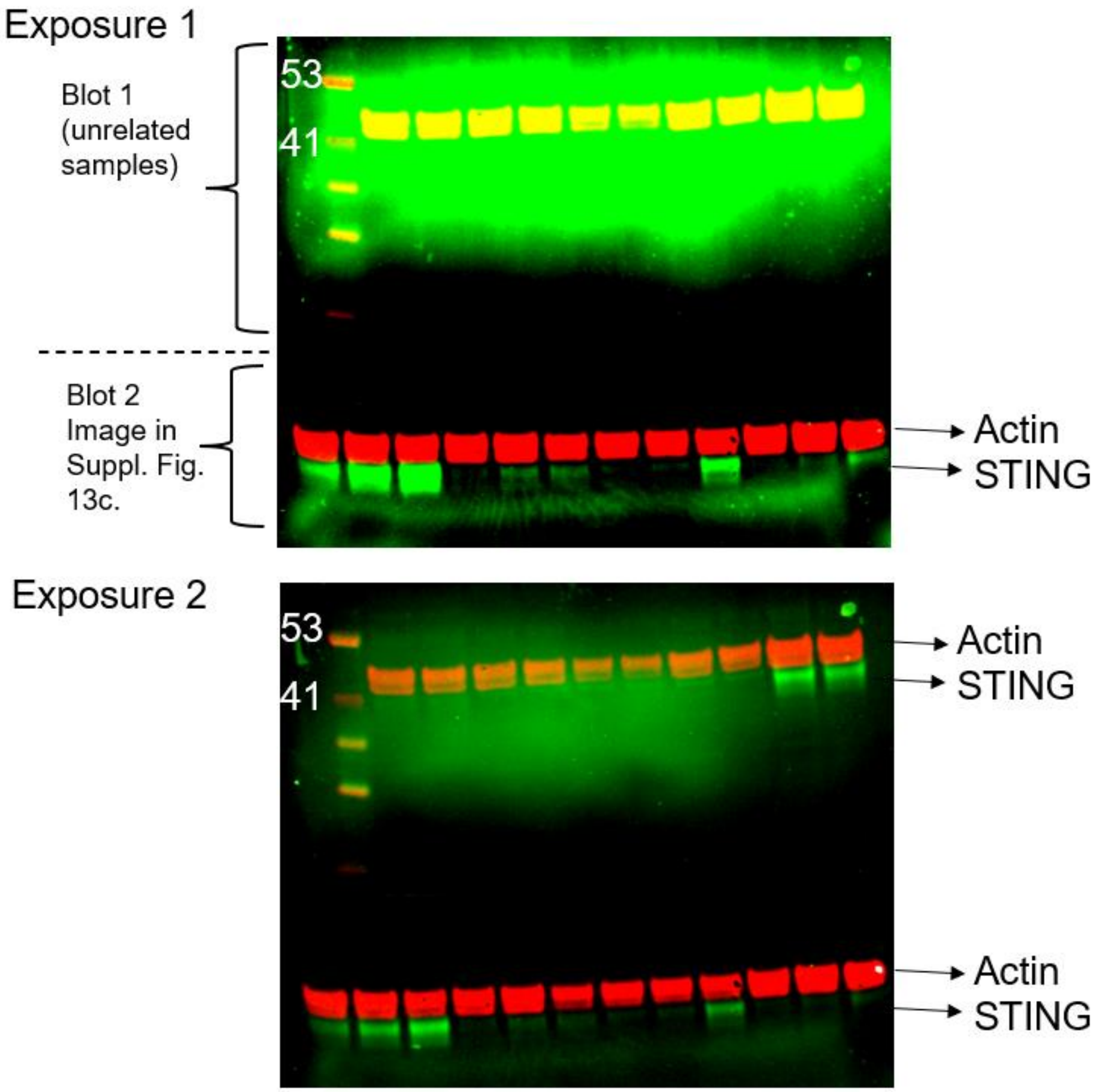

Supplementary Figure 22. Uncropped original scans of the Western blot images in Supplementary Figure 13c.

SUPPLEMENTARY TABLE 1.

Tier 1 characteristics of fresh human tumor tissue samples according to BRISQ reporting guidelines[1]

	Tumor #1	Tumor # 2
Biospecimen Type	Solid tissue, Tumor	Solid tissue, Tumor
Anatomical Site	Ovary	Ovary
Disease status of patients	Ovarian cancer	Ovarian cancer
Clinical characteristics of patients	Unknown	Unknown
Vital State of patients	Alive	Alive
Clinical diagnosis of patients	Ovarian cancer	Ovarian cancer
Pathology diagnosis	Endometrioid carcinoma	Ovarian Serous Carcinoma
Collection mechanism	Surgical resection	Surgical resection
Type of stabilization	Collection media	Collection media
Type of long-term preservation	Not applicable	Not applicable
Constitution of preservative	Not applicable	Not applicable
Storage temperature	+4 to +8 ° C	+4 to +8 ° C
Storage duration	24 hours	24 hours
Shipping temperature	+4 to +8 ° C	+4 to +8 ° C
Composition assessment & selection	Minimum 70% tumor	Minimum 70% tumor

SUPPLEMENTARY REFERENCES

1 Moore, H. M. *et al.* Biospecimen reporting for improved study quality (BRISQ). *Cancer Cytopathol* **119**, 92-101, doi:10.1002/cncy.20147 (2011).
