# Supplementary material for: Tumor cell-directed STING agonist antibody-drug conjugates induce type III interferons and anti-tumor innate immune responses
Source: Nat Commun. 2024 Jul 11;15:5842. doi: 10.1038/s41467-024-49932-4 (PMC11239908; doi:10.1038/s41467-024-49932-4)
Supplement: Supplementary file 3 — Reporting summary [file 41467_2024_49932_MOESM3_ESM.pdf]

nature portfolio

Corresponding author(s): Naniye Cetinbas, Marc Damelin

Last updated by author(s): Mar 25, 2024

Reporting Summary

Nature Portfolio wishes to improve the reproducibility of the work that we publish. This form provides structure for consistency and transparency in reporting. For further information on Nature Portfolio policies, see our Editorial Policies and the Editorial Policy Checklist.

Statistics

For all statistical analyses, confirm that the following items are present in the figure legend, table legend, main text, or Methods section.

n/a	Confirmed	
☐	☒	The exact sample size (*n*) for each experimental group/condition, given as a discrete number and unit of measurement
☐	☒	A statement on whether measurements were taken from distinct samples or whether the same sample was measured repeatedly
☐	☒	The statistical test(s) used AND whether they are one- or two-sided<br>*Only common tests should be described solely by name; describe more complex techniques in the Methods section.*
☒	☐	A description of all covariates tested
☒	☐	A description of any assumptions or corrections, such as tests of normality and adjustment for multiple comparisons
☐	☒	A full description of the statistical parameters including central tendency (e.g. means) or other basic estimates (e.g. regression coefficient) AND variation (e.g. standard deviation) or associated estimates of uncertainty (e.g. confidence intervals)
☐	☒	For null hypothesis testing, the test statistic (e.g. *F*, *t*, *r*) with confidence intervals, effect sizes, degrees of freedom and *P* value noted<br>*Give P values as exact values whenever suitable.*
☒	☐	For Bayesian analysis, information on the choice of priors and Markov chain Monte Carlo settings
☒	☐	For hierarchical and complex designs, identification of the appropriate level for tests and full reporting of outcomes
☒	☐	Estimates of effect sizes (e.g. Cohen's *d*, Pearson's *r*), indicating how they were calculated

Our web collection on statistics for biologists contains articles on many of the points above.

Software and code

Policy information about availability of computer code

Data collection	No custom software was used for data collection in this study other than the softwares in instruments indicated in the manuscript.<br>Instruments used for data collection:<br>nCounter Digital Analyzer Version 3.0.1.4. (Nanostring mRNA data),<br>MACSQuant Analyzer 10,2.11.1817.19623 or Attune NxT Acoustic Focusing Cytometer (flow cytometry data)<br>FlexMAP 3D Luminex Analyzer (Build: 4.2.1513.0) xPONENT software (multiplexed cytokine data)<br>Incucyte Zoom or Incucyte S3 live cell analysis system (live cell imaging/Incucyte data)<br>ForteBio Octet QKe (Octet BLI systems) (Biolayet Interferometry data)<br>Quant Studio 5 rtPCR system (rtPCR data)
Data analysis	No custom software was used for data analysis in this study. All software used were commercial and recommended by the manufacturer of the instruments or the kits/reagents used.<br>nSolver Analysis Software 4.0 Version 2.0.115 (Nanostring mRNA analysis), FlowJo Version 10.8.1 (flow cytometry data analysis), GraphPad Prism Version 9.3.1 or 10.1.2 (data plotting, statistical analysis), Belysa Immuno-Assay Curve-Fitting Software Version 1.0.19 (analysis of cytokine concentrations), ForteBio Analysis Software Version 9 (Biolayer Interferometry data analysis). QuantStudio Design and Analysis software Version 1.5.1 (for rtPCR data analysis), Incucyte ZOOM 2015 Rev1 or Incucyte S3 2020A (Incucyte data analysis), Research Flow Management System (RFMS) Version 2.0.7422 or Study Director Version 3 (animal randomization).

For manuscripts utilizing custom algorithms or software that are central to the research but not yet described in published literature, software must be made available to editors and reviewers. We strongly encourage code deposition in a community repository (e.g. GitHub). See the Nature Portfolio guidelines for submitting code & software for further information.

Data

Policy information about availability of data

All manuscripts must include a data availability statement. This statement should provide the following information, where applicable:

- Accession codes, unique identifiers, or web links for publicly available datasets
- A description of any restrictions on data availability
- For clinical datasets or third party data, please ensure that the statement adheres to our policy

Data is available upon request from the corresponding authors. The source data file of the figures is published alongside this paper.

Research involving human participants, their data, or biological material

Policy information about studies with human participants or human data. See also policy information about sex, gender (identity/presentation), and sexual orientation and race, ethnicity and racism.

Reporting on sex and gender	Study does not involve human participants. Commercial human specimens were purchased/procured without sex, race, ethnicity considerations.
Reporting on race, ethnicity, or other socially relevant groupings	Please see above.
Population characteristics	Please see above.
Recruitment	Please see above.
Ethics oversight	All fresh human tumor tissue samples were appropriately consented and the studies were approved by the IRB (Ohio State Biomedical Institutional Review Board).

Note that full information on the approval of the study protocol must also be provided in the manuscript.

Field-specific reporting

Please select the one below that is the best fit for your research. If you are not sure, read the appropriate sections before making your selection.

☒ Life sciences ☐ Behavioural & social sciences ☐ Ecological, evolutionary & environmental sciences

For a reference copy of the document with all sections, see nature.com/documents/nr-reporting-summary-flat.pdf

Life sciences study design

All studies must disclose on these points even when the disclosure is negative.

Sample size	No statistical analysis was used to predetermine the sample size. Sample sizes were determined based on availability and experimental variability.
Data exclusions	Data was excluded rarely, only when the samples/data failed QC parameters during/after data collection (flagged by instrument or analysis software).
Replication	All experiments were performed using at least 2-4 replicates (biological). All experiments were repeated at different times with consistent observations and findings were verified as indicated in the Methods and Figure legends throughout the manuscript. Serum cytokine, tumor mRNA expression, and PK analyses were performed once using samples from 3-5 different animals. Fresh tumor fragment culture assays were performed once using samples from two different tumors.
Randomization	Mice were randomized into cohorts for all in vivo studies, as indicated in the Methods. RFMS software (v2.0.7422) was used to randomize mice from SKOV3 and 4T1-hHER2 studies. Study Director software was used to randomize OVCAR3 studies by random equilibration.
Blinding	Test article identity was blinded during the conduct of all in vivo studies as well as the fresh tumor fragment culture assays. For the in vitro studies, blinding was not done and was not feasible because the various test articles had different optimal concentration ranges and dilution factors.

Reporting for specific materials, systems and methods

We require information from authors about some types of materials, experimental systems and methods used in many studies. Here, indicate whether each material, system or method listed is relevant to your study. If you are not sure if a list item applies to your research, read the appropriate section before selecting a response.

Materials & experimental systems

n/a	Involved in the study	
☐	☒	Antibodies
☐	☒	Eukaryotic cell lines
☒	☐	Palaeontology and archaeology
☐	☒	Animals and other organisms
☒	☐	Clinical data
☒	☐	Dual use research of concern
☒	☐	Plants

Methods

n/a	Involved in the study	
☒	☐	ChIP-seq
☐	☒	Flow cytometry
☒	☐	MRI-based neuroimaging

Antibodies

Antibodies used	Anti-HER2 antibody Trastuzumab biosimilar was purchased from STC Biologics (cat# STC101). Recombinant anti-NaPi2b human IgG1 (XMT-1535) (DOI: 10.1158/1535-7163.MCT-20-0183) and anti-RSV human IgG1 (Palivizumab) was manufactured by a contract research organization. All flow cytometry antibodies were purchased from Biolegend. PE-anti-FITC antibody (Clone FIT-22, Cat# 408308), PerCP/Cy5.5-CD16 (Clone 3G8, Cat# 302027), PE-CD32 (Clone FUN-2, Cat#303206), APC-Cy7-CD64 (Clone 10.1, Cat# 305025), APC-CD45 (Clone 2D1, Cat#368512), FITC-CD3 (Clone UCHT1, Cat#300406), Pacific Blue-CD14 (Clone 63D3, Cat#367122). Multiple lots were used throughout the study.
Validation	All antibodies used for ADC generation are well-established and validated to bind to their targets with expected affinities using sandwich ELISA-based assays at Mersana Therapeutics. Their purity and quality (lack of aggregation) was confirmed by LC-MS and SEC (size exclusion chromatography). Validation of the Biolegend antibodies are shown on the corresponding product pages on the BioLegend website (traceable from catalog numbers).

Eukaryotic cell lines

Policy information about cell lines and Sex and Gender in Research

Cell line source(s)	Cell Lines Used: HCC1954 (ATCC; CRL-2338), MDA-MB-175-VII (ATCC; HTB-25), Kuramochi (JCRB; #JCRB0098), Calu-3 (ATCC; HTB-55), OVCAR3 (ATCC; HTB-161), THP1-Dual (Invivogen, Cat# thpd-nfis), THP1-Dual KO-STING (Invivogen, Cat# thpd-kostg).<br><br>Primary human PBMCs: Stem Cell Technologies, Cat# 70025.2. Sex was not considered in the studies and the PBMCs were from multiple donors, male or female.
Authentication	Cells were routinely tested for mycoplasma contamination and authenticated using short tandem repeat analysis on a quarterly basis (IDEXX BioAnalytics).
Mycoplasma contamination	Cells were routinely tested for mycoplasma contamination and authenticated using short tandem repeat analysis on a quarterly basis (IDEXX BioAnalytics).
Commonly misidentified lines<br>(See ICLAC register)	None

Animals and other research organisms

Policy information about studies involving animals; ARRIVE guidelines recommended for reporting animal research, and Sex and Gender in Research

Laboratory animals	Species (Mus Muculus) , Female CB17 SCID mice (10 weeks old when SKOV3-tumor bearing mice were dosed, and 17-18 week old when OVCAR3 tumor bearing mice were dosed) and female Balb/c mice (~10 weeks old at the time of dosing) were used in the study.
Wild animals	Study did not involve wild animals.
Reporting on sex	Sex was not considered for the purpose of the studies. Female mice were used for practicality reasons (ie to reduce the chance of fighting when housed in groups)
Field-collected samples	study did not involve samples collected from field.
Ethics oversight	All animal studies were approved by IACUC (Charles River Discovery Services and Translational Drug Development, LLC) and all facilities are AAALAC accredited.

Note that full information on the approval of the study protocol must also be provided in the manuscript.

Plants

Seed stocks: N/A

Novel plant genotypes: N/A

Authentication: N/A

Flow Cytometry

Plots

Confirm that:

☒ The axis labels state the marker and fluorochrome used (e.g. CD4-FITC).

☒ The axis scales are clearly visible. Include numbers along axes only for bottom left plot of group (a 'group' is an analysis of identical markers).

☒ All plots are contour plots with outliers or pseudocolor plots.

☒ A numerical value for number of cells or percentage (with statistics) is provided.

Methodology

Sample preparation: cells were harvested following treatments and transfered to 96 well U-bottom plates, spun down, washed with cold PBS, stained with Aqua viability dye at room temperature for 10 min. Wells were quenched with Flow cytometry staining buffer containing FBS, spun down, washed and resuspended in flow cytometry staining buffer containing fflow cytometry antibodies for ~30 min at RT in dark. Wells were washed 2-3 times with cold PBS and re-suspended in 1-2 % PFA in flow cytometry staining buffer.

Instrument: MACSQuant Analyzer 10,2.11.1817.19623 or Attune NxT Acoustic Focusing Cytometer

Software: All flow cytometry data were analyzed using FlowJo (V 10.8.1) except the data for Napi2b ADC binding to OVCAR3 cells , which was analyzed by MACS Quant Analyzer software.

Cell population abundance: Cell sorting was performed for isolating STING KO cancer cells and CD64 KO THP1 cells in this study. For STING KO cell isolation, single cells were sorted into 96 well tissue culture plates and STING KO was confirmed by Western blot analysis following expansion of the single cell clones. For isolating CD64 KO cells, bulk population of CD64 negative cells (abundance was greater than 50% in all CD64 sgRNA-treated THP1 cells (wt cells are 100% CD64 positive) were sorted. The abundance of CD64+ cells post-sorting was greater than 90%.

Gating strategy: Gating strategy: SSC-A/FSC-A (starting population) --> FSC-H/FSC-A (single cells) --> Aqua (viablity dye)/FSC-A (Single/live cells) --> APC-CD45 / Pac. Blue-EPCAM (CD45+ cells). boundaries between positive and negative populations were defined based on the isotype control-stained and unstained (negative) populations vs fluorophore-target antibody-stained (positive) populations.

☒ Tick this box to confirm that a figure exemplifying the gating strategy is provided in the Supplementary Information.
